# Serum Lipid Abnormalities and Age-Related Cataract: A Hospital-Based Case-Control Study in North India

**DOI:** 10.7759/cureus.117339

**Published:** 2026-10-01

**Authors:** Ruchika Agarwal, Sakshi Bhandari, Pallavi Dhawan, Pawan A Kulkarni, Namrata Prasad, Sadaf Khan

**Affiliations:** 1 Department of Ophthalmology, Rama Medical College Hospital and Research Centre, Kanpur, IND; 2 Department of Biochemistry, Rama Medical College Hospital and Research Centre, Kanpur, IND

**Keywords:** age-related cataract, case control study, dyslipidaemia, hdl cholesterol, ldl cholesterol, serum lipids

## Abstract

Background: Dyslipidaemia may contribute to age-related cataract through altered lens membrane homeostasis, oxidative stress and lipid peroxidation, but clinical evidence remains inconsistent.

Objective: To compare serum lipid profiles in adults with and without age-related cataract and quantify the independent associations of specific lipid abnormalities with cataract.

Methods: This hospital-based case-control study included 300 adults aged 50 years or older at a tertiary care centre in Kanpur, India, from April 2024 to October 2025. Cases were 150 patients with age-related cataract, and controls were 150 participants without cataract. Patients with diabetes, secondary cataract or lipid-lowering therapy were excluded. Fasting total cholesterol, triglycerides, low-density lipoprotein cholesterol (LDL-C), very-low-density lipoprotein cholesterol (VLDL-C) and high-density lipoprotein cholesterol (HDL-C) were measured by enzymatic colourimetry. Separate multivariable logistic regression models adjusted each lipid abnormality for age, sex, education, occupation, smoking, alcohol use and socioeconomic status.

Results: Cases were older than controls (63.07 ± 6.14 vs 60.70 ± 6.67 years; p = 0.002). Compared with controls, cases had higher total cholesterol (209.62 ± 33.23 vs 183.10 ± 28.08 mg/dL), triglycerides (169.42 ± 34.47 vs 146.65 ± 27.12 mg/dL), LDL-C (138.69 ± 30.45 vs 119.17 ± 21.27 mg/dL) and VLDL-C (33.89 ± 6.89 vs 29.33 ± 5.42 mg/dL), and lower HDL-C (39.49 ± 5.90 vs 45.95 ± 6.75 mg/dL; all p < 0.001). Adjusted odds of cataract were higher with low HDL-C (adjusted odds ratio {aOR} 3.91, 95% confidence interval {CI} 2.22-6.89), high LDL-C (aOR 3.06, 95% CI 1.86-5.04), high VLDL-C (aOR 3.03, 95% CI 1.84-5.00), high triglycerides (aOR 2.69, 95% CI 1.64-4.43) and high total cholesterol (aOR 2.49, 95% CI 1.54-4.05). Lipid distributions did not differ significantly across the 15 recorded cataract morphology categories.

Conclusion: Dyslipidaemia, particularly low HDL-C and elevated atherogenic lipids, was independently associated with age-related cataract in this hospital population. These findings identify a clinically relevant metabolic correlate but do not establish causality. Prospective population-based studies should determine whether lipid abnormalities predict cataract onset or progression.

## Introduction

Cataract remains a major cause of blindness and moderate-to-severe vision impairment worldwide, with the greatest burden concentrated in low- and middle-income settings [1]. In India, a nationally representative survey of adults aged 50 years or older found that cataract was the leading cause of blindness and a major contributor to visual impairment [2]. Regional studies also show that age, tobacco exposure, diabetes, hypertension, socioeconomic disadvantage and rural residence influence the occurrence and clinical pattern of age-related cataract [3,4]. Although cataract surgery restores vision, identifying modifiable systemic correlates remains relevant because the demand for surgery continues to rise with population ageing.

Lens transparency depends on the highly ordered arrangement of crystallin proteins and the specialised lipid composition of lens fibre-cell membranes. Human lens membranes contain unusually high concentrations of cholesterol and sphingolipids, which support membrane stability and optical function [5]. Ageing and cataract are accompanied by changes in lipid composition, membrane order and protein-lipid interactions [5,6]. Oxidative modification of lens proteins is a central mechanism in nuclear cataract, while reactive oxygen species can also initiate lipid peroxidation and damage membrane transport systems [7,8]. These observations provide biological plausibility for a relationship between systemic lipid disturbances and lens opacity.

Clinical evidence, however, is heterogeneous. A Chinese case-control study found higher low-density lipoprotein cholesterol (LDL-C), triglyceride and total cholesterol concentrations among patients with age-related cataract, with triglycerides independently associated with disease [9]. The Framingham studies reported longitudinal, sex- and subtype-specific associations between serum lipids and lens opacities [10]. Korean cohort data implicated low high-density lipoprotein cholesterol (HDL-C), while a review of HDL-C in ocular disease concluded that the direction and magnitude of association vary across populations and cataract subtypes [11,12]. A recent meta-analysis of metabolic syndrome and cataract further supports an association between adverse metabolic health and cataract, but also highlights substantial heterogeneity [13].

Evidence from North Indian hospital populations remains limited, and many published studies have not accounted adequately for age and lifestyle covariates. The present study therefore compared serum lipid profiles between adults with age-related cataract and controls without cataract and estimated the adjusted association of individual lipid abnormalities with cataract. A secondary objective was to explore whether serum lipid distributions varied across recorded cataract morphologies.

## Materials and methods

Study design, setting and reporting

A hospital-based case-control study was conducted in the Department of Ophthalmology in collaboration with the Department of Biochemistry at Rama Medical College Hospital and Research Centre, Kanpur, Uttar Pradesh, India. Recruitment and data collection were undertaken from April 2024 to October 2025. The manuscript was prepared in accordance with the Strengthening the Reporting of Observational Studies in Epidemiology (STROBE) statement [14].

Participants and recruitment

Adults aged 50 years or older attending the ophthalmology service were assessed for eligibility. Cases comprised consecutive eligible patients who were clinically diagnosed with age-related cataract by an ophthalmologist. Controls were adults accompanying patients or attending the same hospital who had no clinical evidence of cataract and no history of cataract surgery. Thus, controls were recruited from the same hospital setting as the cases, but were not selected from a population-based sampling frame.

Participants with congenital, traumatic, or secondary cataract, diabetes mellitus, current lipid-lowering therapy, or inability or unwillingness to provide informed consent were excluded. A total of 300 participants were included, comprising 150 cases and 150 controls.

The original study protocol estimated a requirement of approximately 288 participants using an expected cataract prevalence of 25%, 95% confidence, and 5% absolute precision. Recruitment was subsequently set at 300 participants and allocated equally between cases and controls. This was a prevalence-based calculation and was not an effect-size-based sample-size calculation specifically designed for an analytic case-control study. Accordingly, the calculation is reported transparently as a protocol-based recruitment approach rather than as a conventional case-control power calculation.

Clinical and ophthalmic assessment

A structured proforma was used to record age, sex, education, occupation, smoking, alcohol use, socioeconomic status, and clinical data (see Appendices). All participants underwent visual acuity assessment and anterior-segment examination, including slit-lamp biomicroscopy after pharmacological dilation when clinically appropriate.

Cataract was diagnosed clinically by an ophthalmologist. Lens morphology was recorded according to the observed clinical pattern, with nuclear sclerosis graded on a 1-5 scale and the presence or absence of cortical and/or posterior subcapsular components documented. Controls were required to have a clear lens on clinical examination.

The morphology classification used in this study was a clinical grading approach and was not a formally validated photographic grading system such as the Lens Opacities Classification System [15]. Therefore, analyses according to cataract morphology were considered exploratory.

Biochemical measurements and lipid definitions

Venous blood samples were collected after an overnight fast under aseptic conditions. Serum total cholesterol (TC), triglycerides (TG), and HDL-C were measured using enzymatic colourimetric assays on an Erba XL laboratory platform (Erba Mannheim, Germany). LDL-C and very-low-density lipoprotein cholesterol (VLDL-C) were obtained using the laboratory methods specified in the study protocol.

Lipid abnormalities were defined according to Adult Treatment Panel III thresholds [16]: TC ≥200 mg/dL, TG ≥150 mg/dL, LDL-C ≥130 mg/dL, VLDL-C ≥30 mg/dL, and low HDL-C <40 mg/dL in men or <50 mg/dL in women.

Where laboratory documentation was available, assay and quality-control information was reviewed for inclusion in the revised reporting. Details not verifiable from the original study or laboratory records were not retrospectively inferred.

Statistical analysis

Continuous variables were summarised as mean ± standard deviation and categorical variables as number and percentage. Independent-samples t tests were used to compare continuous variables, while Pearson chi-square tests were used for categorical variables. Mean differences were reported with 95% confidence intervals (CIs), and crude odds ratios (ORs) were calculated for each lipid abnormality.

Because the lipid fractions are biologically and statistically correlated, five separate multivariable logistic regression models were fitted. Each model included one lipid abnormality together with the same prespecified measured covariates: age, sex, education, occupation, smoking, alcohol use, and socioeconomic status. Adjusted ORs with 95% CIs were reported.

The separate lipid models were used to reduce the potential effects of correlation among lipid fractions; however, they do not permit determination of whether one lipid fraction is independently associated with cataract after simultaneous adjustment for the other lipid fractions. Accordingly, the resulting estimates are interpreted as associations after adjustment for the measured covariates rather than as independent effects of individual lipid fractions.

The exploratory cataract-morphology analysis summarised lipid concentrations within each morphology group as median and interquartile range (IQR) and compared the 15 morphology groups using the Kruskal-Wallis test. Because several morphology groups were small, these analyses were considered exploratory.

All statistical tests were two-sided, and p<0.05 was considered statistically significant. Original descriptive analyses were performed using IBM SPSS Statistics for Windows, version 21 (IBM Corp., Armonk, NY, USA). Statistical analyses were performed using Python version 3.13 (Python Software Foundation, Beaverton, OR, USA) and statsmodels version 0.14.6 (statsmodels Developers). Core analysis variables were complete for all 300 participants.

The Python re-analysis was conducted using the same prespecified covariates and regression structure described above. 

Ethics

The Institutional Ethics Committee (IEC), Rama Medical College Hospital & Research Centre (RMCHRC), Kanpur, issued approval IEC/RMCHRC/2024/3289A, dated January 27, 2024. Written informed consent was obtained from every participant. The study was conducted in accordance with the principles of the Declaration of Helsinki.

## Results

All 300 enrolled participants were included in the analysis. Cases were older than controls by a mean of 2.37 years (p = 0.002). Sex, education, occupation, smoking, alcohol use and socioeconomic status were not significantly different between groups (Table 1). Because age differed between groups, it was included in every multivariable model. 

**Table 1:** Baseline characteristics of cataract cases and controls. The statistical test used is the chi-square test. Data are presented as mean ± standard deviation or n (%). Percentages for subcategories are calculated within the study group.

Characteristic	Cases (n = 150)	Controls (n = 150)	Test statistic	p value
Age, years	63.07 ± 6.14	60.70 ± 6.67	t = 3.20	0.002
Female sex	66 (44.0)	71 (47.3)	χ² = 0.34	0.562
Education	-	-	χ² = 0.72	0.699
Higher	48 (32.0)	48 (32.0)	-	-
Primary	54 (36.0)	60 (40.0)	-	-
Secondary	48 (32.0)	42 (28.0)	-	-
Occupation	-	-	χ² = 0.29	0.963
Homemaker	17 (11.3)	16 (10.7)	-	-
Retired	8 (5.3)	8 (5.3)	-	-
Skilled	38 (25.3)	42 (28.0)	-	-
Unskilled	87 (58.0)	84 (56.0)	-	-
Current smoking	32 (21.3)	28 (18.7)	χ² = 0.33	0.564
Alcohol use	25 (16.7)	22 (14.7)	χ² = 0.23	0.634
Socioeconomic status	-	-	χ² = 0.63	0.730
High	18 (12.0)	18 (12.0)	-	-
Middle	87 (58.0)	93 (62.0)	-	-
Low	45 (30.0)	39 (26.0)	-	-

Every measured lipid fraction differed between groups (Table 2). Cases had substantially higher total cholesterol, triglycerides, LDL-C and VLDL-C and lower HDL-C. The standardised mean difference was largest for HDL-C (Cohen d = -1.02), followed by total cholesterol (d = 0.86), indicating large between-group separation for these measures.

**Table 2:** Comparison of serum lipid concentrations between cases and controls. Values are mean ± standard deviation. Between-group comparisons used independent-samples t tests (df = 298) as the statistical test. Reference ranges denote desirable clinical concentrations based on Adult Treatment Panel III [16] thresholds. CI, confidence interval; HDL-C, high-density lipoprotein cholesterol; LDL-C, low-density lipoprotein cholesterol; VLDL-C, very-low-density lipoprotein cholesterol. The mean difference is cases minus controls.

Lipid parameter	Reference range	Cases (n = 150)	Controls (n = 150)	Test statistic	Mean difference (95% CI)	Cohen d	p value
Total cholesterol	<200 mg/dL	209.62 ± 33.23	183.10 ± 28.08	t = 7.47	26.52 (19.53 to 33.51)	0.86	<0.001
Triglycerides	<150 mg/dL	169.42 ± 34.47	146.65 ± 27.12	t = 6.36	22.78 (15.73 to 29.83)	0.73	<0.001
LDL-C	<130 mg/dL	138.69 ± 30.45	119.17 ± 21.27	t = 6.44	19.52 (13.55 to 25.49)	0.74	<0.001
VLDL-C	<30 mg/dL	33.89 ± 6.89	29.33 ± 5.42	t = 6.37	4.55 (3.15 to 5.96)	0.73	<0.001
HDL-C	≥40 men; ≥50 women	39.49 ± 5.90	45.95 ± 6.75	t = −8.83	−6.46 (−7.90 to −5.02)	−1.02	<0.001

The prevalence of each adverse lipid category was higher among cases. Crude ORs ranged from 2.38 for high triglycerides to 2.90 for low HDL-C. After adjustment for age and sociodemographic and lifestyle variables, all five associations remained statistically significant (Table 3). Low HDL-C showed the strongest adjusted association, with nearly fourfold higher odds of cataract. The adjusted estimates are displayed in Figure 1. 

**Table 3:** Crude chi-square and multivariable logistic regression associations between lipid abnormalities and age-related cataract. Total sample: 150 cases and 150 controls. Crude p values were obtained using Pearson chi-square tests. Each adjusted odds ratio was estimated in a separate multivariable binary logistic regression model containing one lipid abnormality and the covariates age, sex, education, occupation, smoking, alcohol use and socioeconomic status. Reference categories were: total cholesterol <200 mg/dL; triglycerides <150 mg/dL; LDL-C <130 mg/dL; VLDL-C <30 mg/dL; and HDL-C ≥40 mg/dL in men or ≥50 mg/dL in women. CI, confidence interval; OR, odds ratio; HDL-C, high-density lipoprotein cholesterol; LDL-C, low-density lipoprotein cholesterol; VLDL-C, very-low-density lipoprotein cholesterol.

Lipid abnormality	Cases n (%)	Controls n (%)	Crude OR (95% CI)	Crude p	Adjusted OR (95% CI)	Adjusted p
Total cholesterol ≥200 mg/dL	92 (61.3)	58 (38.7)	2.52 (1.58–4.00)	<0.001	2.49 (1.54–4.05)	<0.001
Triglycerides ≥150 mg/dL	88 (58.7)	56 (37.3)	2.38 (1.50–3.79)	<0.001	2.69 (1.64–4.43)	<0.001
LDL-C ≥130 mg/dL	85 (56.7)	49 (32.7)	2.70 (1.69–4.31)	<0.001	3.06 (1.86–5.04)	<0.001
VLDL-C ≥30 mg/dL	93 (62.0)	56 (37.3)	2.74 (1.72–4.37)	<0.001	3.03 (1.84–5.00)	<0.001
Low HDL-C	113 (75.3)	77 (51.3)	2.90 (1.77–4.72)	<0.001	3.91 (2.22–6.89)	<0.001

The Kruskal-Wallis analysis showed no statistically significant differences in serum lipid distributions across the 15 cataract morphology categories (n = 150). The 150 cases were distributed across 15 recorded morphology categories combining nuclear sclerosis grades 1 to 5 with the presence or absence of cortical and posterior subcapsular components. The most frequent individual category was nuclear sclerosis grade 4 without an additional component (28/150, 18.7%), followed by grade 3 (23/150, 15.3%) and grade 2 (18/150, 12.0%). Table 4 presents all 15 morphology groups, the number of cases in each group, and the serum lipid values in each group as median (IQR) in mg/dL, together with the corresponding Kruskal-Wallis comparisons. Although total cholesterol demonstrated a comparatively stronger trend toward heterogeneity, the association remained statistically nonsignificant at the conventional α = 0.05 level.

**Table 4:** Serum lipid values across 15 cataract morphology groups with Kruskal-Wallis comparisons (n = 150 cases). Values are median (interquartile range (IQR)) and are expressed in milligrams per deciliter (mg/dL). Kruskal-Wallis tests compared all 15 morphology groups; degrees of freedom (df) = 14 for each test. For total cholesterol, H = 21.25, p = 0.095; for triglycerides, H = 9.85, p = 0.773; for LDL-C, H = 10.23, p = 0.745; for VLDL-C, H = 9.65, p = 0.787; and for HDL-C, H = 17.05, p = 0.254. H, Kruskal-Wallis H statistic; IQR, interquartile range; df, degrees of freedom; NS1, nuclear sclerosis grade 1; NS2, nuclear sclerosis grade 2; NS3, nuclear sclerosis grade 3; NS4, nuclear sclerosis grade 4; NS5, nuclear sclerosis grade 5; PSC, posterior subcapsular cataract; HDL-C, high-density lipoprotein cholesterol; LDL-C, low-density lipoprotein cholesterol; VLDL-C, very-low-density lipoprotein cholesterol; mg/dL, milligrams per deciliter. No lipid parameter differed significantly across morphology groups.

Cataract morphology group	n	Total cholesterol (mg/dL)	Triglycerides (mg/dL)	LDL-C (mg/dL)	VLDL-C (mg/dL)	HDL-C (mg/dL)
NS1	6	206.2 (187.6-230.1)	180.2 (149.9-209.9)	139.3 (117.9-163.6)	36.0 (30.0-42.0)	38.6 (34.9-44.9)
NS1 + Cortical	6	208.4 (193.3-232.9)	164.0 (149.3-182.6)	120.5 (111.8-124.0)	32.8 (29.9-36.5)	34.7 (32.5-35.3)
NS1 + PSC	3	228.8 (210.2-237.3)	144.8 (140.1-168.8)	147.6 (147.0-159.3)	29.0 (28.1-33.8)	39.1 (37.9-42.2)
NS2	18	193.6 (177.9-214.9)	190.2 (175.6-195.8)	128.3 (116.7-158.8)	38.0 (35.1-39.2)	39.9 (35.0-43.5)
NS2 + Cortical	15	217.3 (193.7-223.5)	166.5 (146.7-188.1)	139.2 (111.8-167.6)	33.3 (29.4-37.6)	44.2 (38.0-46.1)
NS2 + PSC	4	238.1 (230.4-244.6)	149.2 (138.1-162.7)	136.0 (111.2-159.6)	29.9 (27.6-32.5)	38.8 (32.5-44.3)
NS3	23	201.3 (182.3-222.4)	179.0 (149.3-201.8)	137.9 (124.3-161.2)	35.8 (29.9-40.4)	38.9 (35.5-42.5)
NS3 + Cortical	12	224.7 (190.3-264.7)	149.2 (142.1-182.5)	157.4 (141.9-168.9)	29.9 (28.4-36.5)	38.5 (34.9-43.6)
NS3 + PSC	10	201.1 (194.4-209.2)	167.6 (149.3-176.1)	148.3 (114.2-156.4)	33.5 (29.8-35.2)	39.8 (34.1-43.6)
NS4	28	215.5 (188.7-230.8)	171.7 (148.6-183.3)	132.5 (115.8-148.2)	34.3 (29.7-36.6)	37.4 (34.6-39.6)
NS4 + Cortical	6	213.8 (209.6-216.0)	165.2 (136.1-193.1)	148.4 (137.1-160.2)	33.0 (27.2-38.6)	39.5 (38.9-41.5)
NS4 + PSC	3	275.9 (248.9-281.8)	170.3 (148.1-173.9)	135.4 (113.2-138.7)	34.1 (29.6-34.8)	42.8 (41.1-43.8)
NS5	10	181.6 (176.6-199.5)	174.0 (128.6-199.3)	141.0 (123.2-157.0)	34.8 (25.7-39.9)	38.9 (38.1-39.7)
NS5 + Cortical	3	195.4 (176.2-215.7)	180.7 (180.5-181.8)	149.1 (119.8-159.4)	36.1 (36.1-36.4)	41.7 (37.0-42.7)
NS5 + PSC	3	226.0 (211.5-233.2)	187.1 (146.9-212.4)	135.9 (132.4-143.3)	37.4 (29.4-42.5)	47.8 (42.3-48.7)

## Discussion

In this hospital-based case-control study, age-related cataract was associated with a consistent adverse lipid profile. Cases had higher total cholesterol, triglycerides, LDL-C and VLDL-C and lower HDL-C than controls. These differences were not only statistically significant but also clinically substantial, with standardised mean differences ranging from 0.73 to 1.02 in absolute magnitude. After adjustment for age, sex and measured social and lifestyle variables, all five binary lipid abnormalities remained associated with cataract. Low HDL-C showed the largest adjusted OR, followed by elevated LDL-C and VLDL-C. No robust subtype-specific pattern emerged across the detailed morphology categories.

The direction of the present findings is broadly consistent with the Chinese study by Li et al., which reported higher LDL-C, triglycerides and total cholesterol among 219 cataract cases than among 218 controls and identified triglycerides as an independent correlate [9]. Our adjusted effect estimates also agree with reports linking low HDL-C to cataract in Korean adults [11]. The magnitude of the low-HDL association in this study is stronger than that reported in several population-based datasets, but falls within the wide range summarised in the review by Betzler et al. [12]. Differences in participant selection, sex distribution, age, diet, ethnicity, lipid thresholds and control of confounding may explain between-study variation.

The Framingham investigation identified sex- and subtype-dependent associations, including an association between persistent hypertriglyceridaemia and posterior subcapsular opacity in men [10]. In contrast, the current exploratory analysis did not find significant differences in lipid distributions across 15 morphologic categories. The absence of subtype differences should be interpreted cautiously because many categories were sparse and the classification combined nuclear grade with cortical or posterior subcapsular components. Larger studies using a standardized grading system, such as the Lens Opacities Classification System III (LOCS III), are needed for reliable subtype inference [15].

The broader metabolic literature also supports a relationship between cataract and adverse metabolic health. A 2025 meta-analysis of 10 observational studies and 379,464 participants found that metabolic syndrome was associated with a 28% higher cataract risk, although heterogeneity was high [13]. Earlier studies from Lithuania, Singapore, Australia and Korea have produced mixed results for individual lipid components [12,17-19]. These inconsistencies make it unlikely that a single circulating lipid fraction explains cataractogenesis in all populations. Rather, dyslipidaemia may act as one component of a broader metabolic and oxidative phenotype. Using two-sample Mendelian randomisation, genetically determined primary open-angle glaucoma and myopic refractive error were significantly associated with cataract risk (POAG: OR 1.04, 95% CI 1.01-1.08, P = 0.018; per diopter more hyperopic: OR 0.92, 95% CI 0.89-0.93, P = 6.51 × 10⁻¹³), and this POAG association persisted after adjusting for refractive error. In contrast, genetically determined type 2 diabetes, blood pressure, body mass index, cigarette smoking, and alcohol consumption were not associated with cataract risk, suggesting that, despite consistent observational links, these behavioural and metabolic factors may not be independently causal [20].

Several mechanisms could account for the observed associations. The lens possesses a highly specialised membrane lipid composition, and age-related changes in cholesterol and sphingolipid organisation influence membrane order and protein interactions [5,6]. Elevated circulating atherogenic lipids may increase systemic oxidative stress and lipid peroxidation. Reactive aldehydes generated during lipid peroxidation can modify crystallins, damage lens epithelial cells and impair membrane transport [8]. Oxidative modification and aggregation of long-lived lens proteins are central to nuclear cataract formation [7].

HDL-C may be particularly relevant because HDL participates in reverse cholesterol transport and carries antioxidant and anti-inflammatory enzymes. Low HDL-C could therefore indicate reduced systemic capacity to neutralise oxidised lipids. Conversely, high LDL-C and triglycerides may increase the pool of oxidation-prone lipoproteins. The strong association with low HDL-C in the present study is biologically plausible, but serum HDL-C concentration is an imperfect surrogate for HDL particle function [12]. Direct measures of oxidised LDL, apolipoproteins, paraoxonase activity and lens oxidative biomarkers would provide stronger mechanistic evidence.

Strengths include a balanced case-control sample, consecutive recruitment of eligible cases, standardised clinical ophthalmic assessment, measurement of a complete conventional lipid profile, exclusion of diabetes and lipid-lowering treatment, and re-analysis of the participant-level dataset using multivariable models. Reporting both continuous differences and adjusted categorical associations improves clinical interpretability. Separate regression models were used because of the biological and statistical correlation among lipid fractions. Several limitations require emphasis. First, the hospital-based design may produce selection bias, and controls recruited from attendants or hospital visitors may not represent the source population that generated the cases. Second, the original sample-size calculation was prevalence-based rather than an effect-size-based calculation for an analytic case-control design; therefore, the study should not be interpreted as having been powered using a conventional case-control sample-size framework. Third, cases were older than controls, indicating that individual age matching was not achieved. Statistical adjustment reduces but does not eliminate residual confounding. Fourth, body mass index, waist circumference, dietary intake, ultraviolet exposure, physical activity, hypertension, steroid use and antioxidant status were not available for multivariable adjustment. Fifth, the cataract morphology classification was based on clinical assessment and was not a formally validated photographic grading system; consequently, morphology-specific findings should be regarded as exploratory, particularly because several subgroups were sparse. Sixth, a single lipid measurement cannot establish long-term exposure or temporal sequence, and reverse causation and unmeasured illness remain possible. Seventh, exclusion of diabetes improves internal focus but limits generalisability because diabetes is common among older adults with cataract. Eighth, because lipid fractions were analysed in separate regression models, the results cannot determine which correlated lipid component has an association independent of the other lipid fractions. Finally, the study was conducted at one centre in Kanpur, and external validity to other populations is uncertain.

Several limitations require emphasis. First, the hospital-based design may produce selection bias, and controls recruited from attendants or hospital visitors may not represent the source population that generated the cases. Second, cases were older than controls, indicating that individual age matching was not achieved. Statistical adjustment reduces but does not eliminate residual confounding. Third, body mass index, waist circumference, dietary intake, ultraviolet exposure, physical activity, hypertension, steroid use and antioxidant status were not available for multivariable adjustment. Fourth, a single lipid measurement cannot establish long-term exposure or temporal sequence. Reverse causation and unmeasured illness remain possible. Fifth, exclusion of diabetes improves internal focus but limits generalisability because diabetes is common among older adults with cataract. Sixth, detailed cataract categories were sparse, limiting power for subtype comparisons. Finally, the study was conducted at one centre in Kanpur, and external validity to other populations is uncertain.

## Conclusions

Adults with age-related cataract had higher total cholesterol, triglycerides, LDL-C and VLDL-C and lower HDL-C than controls. Each lipid abnormality remained independently associated with cataract after adjustment for age and measured sociodemographic and lifestyle factors, with the strongest association observed for low HDL-C. The results support dyslipidaemia as a clinically relevant correlate of age-related cataract but cannot establish causality. Prospective multicentre studies are needed to determine whether serum lipid patterns predict cataract development, progression or subtype.

## Appendices

Questionnaire

Title: “To Study the Association of Serum Lipid Levels with Age-Related Cataract: A Case-Control Study”

•        Name:…………………………  Age (years):…………  Gender:…………

•        Past medical history:

•        Duration of Cataract (years):

•        Comorbid Conditions: HTN/DM/COPD/others

•        Systolic and Diastolic Blood Pressure (mmHg):

•        Random blood sugar(mg/dl)

•        Addiction: Alcohol/Tobacco/Smoking/others

•        Diet: Vegetarian/Non-vegetarian

•        Visual Acuity Assessment

•        Intraocular Pressure (mmHg)

•        Anterior Segment Examination

•        Grading of Cataract

•        Fundus Examination using

       Indirect ophthalmoscope

       Slit lamp with 90D

       Fundus picture

•        Lipid Profile

v Total Cholesterol

v HDL Cholesterol

v LDL

v VLDL

v Triglyceride

v Cholesterol/HDL

v LDL/HDL
